# Case Report: An Incidental Finding of Fahr's Disease in a Patient with Hypochondria

**DOI:** 10.7759/cureus.3668

**Published:** 2018-12-01

**Authors:** Courtland R Samuels, Javed L Khanni, Kory Barkley, Mishah Azhar, Patricio S Espinosa

**Affiliations:** 1 Emergency Medicine, Florida Atlantic University Charles E. Schmidt College of Medicine, Boca Raton, USA; 2 Neurology, Florida Atlantic University Charles E. Schmidt College of Medicine, Boca Raton, USA; 3 Internal Medicine, Florida Atlantic University Charles E. Schmidt College of Medicine, Boca Raton, USA; 4 Neurology, Marcus Neuroscience Institute, Boca Raton, USA

**Keywords:** fahr's disease, basal ganglia calcification, bilateral striopallidodentate calcinosis, movement disorder, hypochondria, slc20a2, pdgfrb

## Abstract

Idiopathic basal ganglia calcification (IBGC), commonly referred to as Fahr’s disease, is a rare neurological disorder characterized by the abnormal, symmetrical, and bilateral calcification of the basal ganglia and other brain regions. Patients typically present in their forties and fifties with various neurologic and/or psychiatric symptoms, including movement disorders, Parkinsonism, psychosis, and depression.

The pathophysiology of this disease is not completely understood; however, several gene mutations have been identified in the pathogenesis of Fahr’s disease. These mutations display an autosomal dominant inheritance pattern. Furthermore, the regional phenotypic expression of calcifications differs greatly from patient to patient, as do their clinical presentations. Here, we describe a patient who presented with psychiatric manifestations and imaging consistent with Fahr’s disease.

## Introduction

Fahr’s disease, also known as idiopathic basal ganglia calcification (IBGC), or bilateral striopallidodentate calcinosis, is a condition characterized by calcium deposition in the brain, most often the basal ganglia. Typical presentation occurs in early or middle adulthood with neurologic or psychiatric features [1]. A wide variety of symptoms have been documented, with Parkinsonism being the most common [2]. Other reported symptoms include cognitive impairment, ataxia, dysarthria, tremor, seizures, hallucinations, delusions, anxiety, and depression [1]. A computed tomography (CT) scan of the brain is the most useful aid in diagnosis and typically reveals bilateral calcification in the basal ganglia. Other structures commonly involved include the dentate nucleus, thalamus, and white matter [3]. Magnetic resonance imaging (MRI) has been shown to display T2-weighted hyperintensities that are believed to be related to an underlying progressive metabolic or inflammatory process. It has been suggested that the progressive processes are responsible for the neurological features seen in the disease, and the calcification typically noted on a CT scan is a later consequence [4]. Familial and non-familial forms of Fahr’s disease exist, with familial forms inherited in an autosomal dominant fashion [1]. Although the mechanism has yet to be elucidated, a number of genes have been identified, including *SLC20A2 *and *PDGFRB *[5-6]. Here, we describe the case of a patient who presented with psychiatric manifestations, leading to the incidental diagnosis of Fahr's disease.

## Case presentation

A 47-year-old Caucasian male with a past medical history of uncontrolled diabetes mellitus, hypertension, gastroesophageal reflux disease, and anxiety presented to the emergency department with a chief complaint of right foot swelling. The patient reported that the swelling started two weeks prior to the presentation but denied any associated pain. The patient denied injury or trauma to the foot. An X-ray of the right foot revealed old fracture deformities but no evidence of an acute osseous lesion. A lower extremity Doppler ultrasound was performed and ruled out the presence of deep vein thrombosis (DVT). The patient also endorsed dysuria, dribbling, and urinary retention over the previous day but adamantly refused placement of a catheter. When the patient was told that he would be discharged from the emergency department, he began complaining of chest tightness, shortness of breath, and nausea, all of which he denied on a review of symptoms during the initial evaluation. He stated that the chest discomfort had been present all day, was non-radiating, and rated at a 4/10 in severity. Electrocardiography (EKG) and cardiac enzymes were within normal limits. The patient was admitted for further evaluation.

During the hospitalization, the patient developed multiple additional complaints that after appropriate workup, ultimately did not lead to a specific diagnosis. The patient worked with physical therapy and occupational therapy, which established that the patient was difficult to assess, as his functional mobility issues were inconsistent. He was noted to have deficits in balance, endurance, and safety awareness, which affected the patient’s ability to perform the activities of daily living. Of note, one of the physical therapists documented that the patient was witnessed alone in his room, walking without difficulty while texting on his phone.

**Figure 1:**
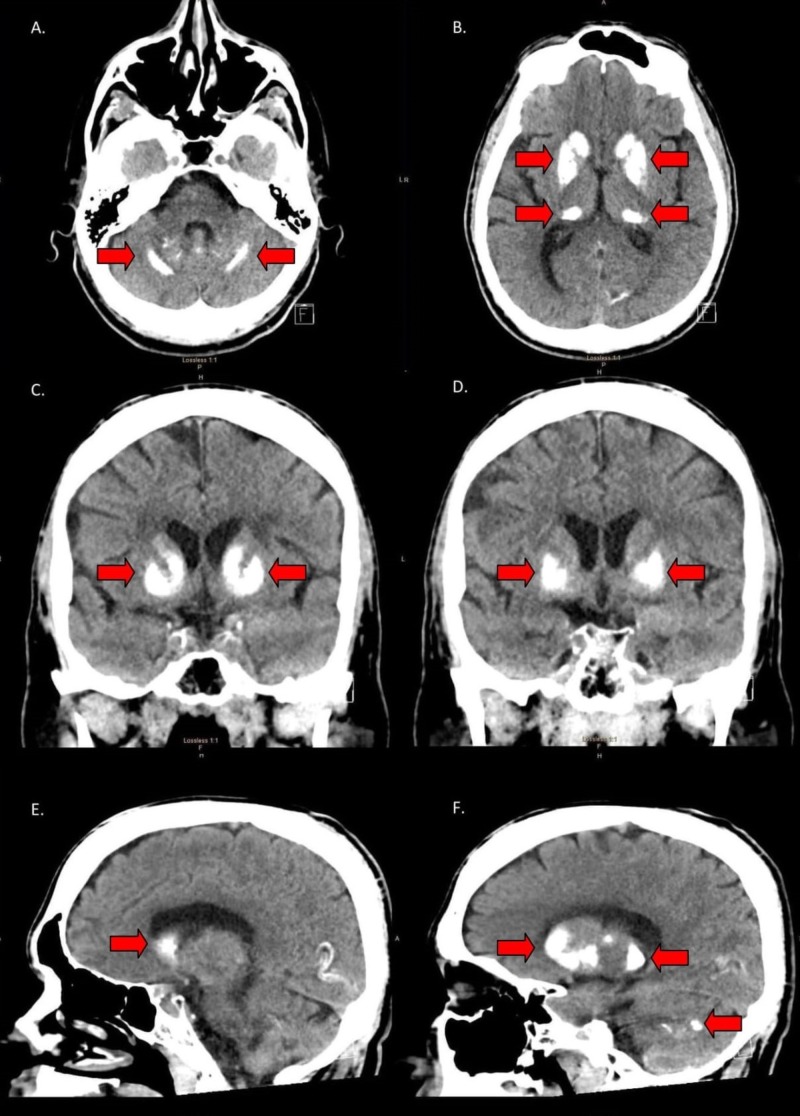
CT images displaying calcifications of the basal ganglia and cerebellum A. Axial CT image showing bilateral, linear calcifications of the cerebellum. B. Axial CT image showing bilateral calcifications of the putamen and the head and tail of the caudate nucleus. C. Coronal CT image showing bilateral calcifications of the caudate nucleus and putamen. D. Coronal CT image showing bilateral calcifications of the putamen. E. Sagittal CT image showing calcification of the head of the caudate nucleus. F. Sagittal CT image showing calcifications throughout the basal ganglia and cerebellum. CT: computed tomography

On hospital day four, the patient was displeased when told that he was being discharged, as he desired further workup for his complaints. While preparing to leave, he had an unwitnessed fall in his room. The patient stated that he hit his head and was in pain but was unable to localize the pain. There was no evidence of acute trauma on physical exam. Regardless, the fall prompted a computed tomography (CT) scan of the brain without contrast to rule out any acute intracranial trauma. Incidentally, the CT scan revealed bilateral symmetric calcifications of the basal ganglia, putamen, caudate, thalami, dentate nuclei of the cerebellum, and cerebral white matter, consistent with Fahr's disease (Figure 1). Laboratory values, such as parathyroid hormone (PTH), thyroid stimulating hormone (TSH), T3, and T4, were all within the normal reference limits. Serum calcium was mildly decreased, ranging from 8.1 - 8.4 mg/dL during the admission. After being informed of the CT scan findings, the patient revealed that his deceased mother suffered from a rare diagnosis. Further investigation, with the patient's permission, revealed a prior CT scan with similar calcifications in the basal ganglia and cerebellum, consistent with Fahr's disease.

## Discussion

This patient’s presentation met the necessary criteria for the diagnosis of Fahr’s disease (Table 1). Specifically, he had a family history of Fahr’s disease, bilateral calcifications of the basal ganglia on neuroimaging, and an absence of other potential etiologies. What makes our patient’s presentation unique is that he exhibited few of the commonly documented manifestations seen in the literature. At the age of 47, we would expect him to present with movement disorders, as they are found in the majority of patients, with Parkinsonism being the most common [1-2]. Other movement manifestations that have been reported include chorea, tremor, dystonia, paresis, athetosis, ataxia, and dysarthria [1]. Although the patient was found to have gait abnormalities, when evaluated by the physical therapist, they were noted to be inconsistent with any known movement disorder and present only intermittently. Furthermore, nursing staff reported seeing him walking freely with a normal gait and without the use of any assistance devices. This raised questions as to whether or not he truly had any of the physical symptoms of which he complained. In 40% of Fahr’s disease cases, the first presenting feature of basal ganglia calcifications is psychiatric manifestations [1]. Depression and anxiety are the most common psychiatric findings associated with Fahr’s disease [7]. Others that have been reported in the literature include dementia, delirium, confusion, hallucinations, delusions, catatonia, mania, panic attacks, obsessive behaviors, irritability, aggression, personality disorder, and personality changes [1].

**Table 1:** Criteria for the diagnosis of Fahr’s disease In the presence of a positive family history (criterion six), the diagnosis can be made in the absence of bilateral calcification (criterion one) or progressive neurologic dysfunction and neuropsychiatric manifestations (criterion two).

Criteria for the diagnosis of Fahr’s disease	
1	Bilateral calcification of the basal ganglia on neuroimaging or other brain regions
2	Progressive neurological dysfunction and/or neuropsychiatric manifestations
3	Age of onset is typically the fourth or fifth decade, may be present also earlier in life
4	Absence of biochemical abnormalities and somatic features suggestive of a mitochondrial or metabolic disease or other systemic disorder
5	Absence of an infectious, toxic, or traumatic cause
6	Family history consistent with autosomal dominant inheritance

Upon presentation and during his hospital stay, our patient had multiple, fluctuating complaints. Laboratory and imaging studies completed during his admission revealed no abnormalities that could explain the patient’s multiple complaints. Furthermore, he participated in behaviors that would directly skew his lab results, in turn, prolonging his hospital stay. Retrospectively, these occurrences have led us to believe that the patient’s complaints and gait abnormalities were possibly factitious in nature and, potentially, the result of a neuropsychiatric manifestation of Fahr’s disease. The patient presented in this case had a previous, self-reported diagnosis of anxiety, which was not confirmed with an evaluation by a psychiatrist during this admission. However, he exhibited several behaviors that could suggest an alternate and/or coexisting psychiatric diagnosis, such as somatic symptom disorder, factitious disorder, or malingering.

Although sporadic cases of Fahr’s disease abound, there seems to exist an autosomal dominant inheritance pattern amongst families with this disease. There are two genes that predominate in the literature, which mutations are associated with Fahr’s disease: SLC20A2 and PDGFRB [5-6]. There are different theories regarding the genetic etiology of Fahr’s disease. Mutations in the SLC20A2 gene encoding the inorganic phosphate transporter (PiT2) can explain approximately 40% of the familial cases of Fahr’s disease. It is believed that a mutation of PiT2 disrupts cellular calcium and phosphate homeostasis, leading to the deposition of calcium phosphate. The PDGFRB gene, as well as the *PDGFB *gene, are believed to be involved in the maintenance of the blood-brain barrier. Mutations lead to alterations in permeability, resulting in calcium deposition. A third gene gaining popularity, XPR1, is also related to PiT2, as it encodes a receptor specifically involved in phosphate homeostasis. Similarly to SLC20A2, mutations in XPR1 lead to calcium deposition [8].

Phenotypically, the regional location and quantity of calcifications vary greatly among patients with Fahr’s disease. In a study looking to accurately diagnose and describe the clinical and radiological characteristics of Fahr’s disease, researchers found that each mutated gene was associated with a different pattern of calcifications, though there was no significant difference in the clinical presentation based on the gene affected. Specifically, patients with the SLC20A2 mutation had more severe calcifications overall and in the lenticular, caudate, and subcortical white matter. Patients with the PDGFRB mutation did not show any cortical or vermis calcifications, contrary to patients with the SLC20A2 mutation, of which 77% showed cortical calcifications and 77% showed vermis calcifications. Furthermore, they found that the severity of calcification correlated positively with age and that those who were symptomatic had a higher total calcification score than those who were asymptomatic. However, the quantification of calcifications seemed insufficient to predict clinical severity; likewise, the localization of calcifications did not significantly correlate with neurological/psychiatric symptomatology [9].

Looking at this study, we propose that symptoms seen in Fahr’s disease are more likely to be a direct result of the underlying pathophysiologic process rather than the specific gene affected or the amount/location of calcifications. This is consistent with the MRI study completed by Avraham et al. [4]. It can then be reasoned that the affected genes lead to a pathophysiologic process that underscores both the calcifications and neuropsychiatric manifestations seen in Fahr’s disease. To further elucidate this theory, more studies need to be completed on the pathophysiology of Fahr’s disease. Potential studies using animal models could be completed to look at the metabolic, physiologic, and/or biologic effects of the deranged protein products associated with the already identified gene mutations.

## Conclusions

Fahr’s disease is characterized by bilateral abnormal calcifications in the brain, most commonly the basal ganglia. Manifestations typically appear in the fourth or fifth decade of life, with movement disorders (most commonly Parkinsonism) and/or neuropsychiatric symptoms (most commonly depression and anxiety). Our case report highlights an unusual presentation of Fahr’s disease in a patient who was seemingly asymptomatic but may, in fact, have psychiatric manifestations related to his disease process. Typically inherited in an autosomal dominant fashion, there have been several genes identified that are associated with Fahr’s disease. These include *SLC20A2*, *PDGFRB*, *PDGFB, *and *XPR1*. Barring further investigation, the clinical significance of these gene mutations, as well as potential underlying pathophysiologic processes, have yet to be identified.
